# CD4 T Cell Determinants in West Nile Virus Disease and Asymptomatic Infection

**DOI:** 10.3389/fimmu.2020.00016

**Published:** 2020-01-23

**Authors:** Maximilian Koblischke, Felicia S. Spitzer, David M. Florian, Stephan W. Aberle, Stefan Malafa, Ingrid Fae, Irene Cassaniti, Christof Jungbauer, Bernhard Knapp, Hermann Laferl, Gottfried Fischer, Fausto Baldanti, Karin Stiasny, Franz X. Heinz, Judith H. Aberle

**Affiliations:** ^1^Center for Virology, Medical University of Vienna, Vienna, Austria; ^2^Department of Blood Group Serology and Transfusion Medicine, Medical University of Vienna, Vienna, Austria; ^3^Molecular Virology Unit, Microbiology and Virology Department, Fondazione IRCCS Policlinico San Matteo, Pavia, Italy; ^4^Department of Clinical, Surgical, Diagnostic and Pediatric Sciences, University of Pavia, Pavia, Italy; ^5^Blood Service for Vienna, Lower Austria and Burgenland, Austrian Red Cross, Vienna, Austria; ^6^Data Science Section, Symptoma GmbH, Vienna, Austria; ^7^Sozialmedizinisches Zentrum Süd, Kaiser-Franz-Josef-Spital, Vienna, Austria

**Keywords:** West Nile virus, flavivirus, CD4 T cell, immunodominance, epitope, West Nile patients

## Abstract

West Nile (WN) virus infection of humans is frequently asymptomatic, but can also lead to WN fever or neuroinvasive disease. CD4 T cells and B cells are critical in the defense against WN virus, and neutralizing antibodies, which are directed against the viral glycoprotein E, are an accepted correlate of protection. For the efficient production of these antibodies, B cells interact directly with CD4 helper T cells that recognize peptides from E or the two other structural proteins (capsid-C and membrane-prM/M) of the virus. However, the specific protein sites yielding such helper epitopes remain unknown. Here, we explored the CD4 T cell response in humans after WN virus infection using a comprehensive library of overlapping peptides covering all three structural proteins. By measuring T cell responses in 29 individuals with either WN virus disease or asymptomatic infection, we showed that CD4 T cells focus on peptides in specific structural elements of C and at the exposed surface of the pre- and postfusion forms of the E protein. Our data indicate that these immunodominant epitopes are recognized in the context of multiple different HLA molecules. Furthermore, we observed that immunodominant antigen regions are structurally conserved and similarly targeted in other mosquito-borne flaviviruses, including dengue, yellow fever, and Zika viruses. Together, these findings indicate a strong impact of virion protein structure on epitope selection and antigenicity, which is an important issue to consider in future vaccine design.

## Introduction

West Nile (WN) virus is a mosquito-borne flavivirus that has caused repeated epidemics in the Americas since its first appearance in 1999, with more than 48,000 documented human cases and ~2,300 deaths (1). In addition, several recent WN virus outbreaks have been reported in Europe, the largest in 2018, involving over 2,000 human cases in 15 countries (1–3). Although most WN virus infections remain asymptomatic (4), about 20% of humans develop symptoms of WN fever, and about one in 150 develop acute neuroinvasive disease (5). Despite significant disease burden, there is no specific treatment or vaccine against WN virus licensed for human use. Protective immunity to WN virus has been mainly attributed to neutralizing antibodies, which are essential for the clearance of infection and long-term immunity against disease (6–8). CD4 T cells also play critical roles in the defense against WN virus, both by promoting protective antibody responses and by direct killing of infected cells (9, 10). Despite this central role, the factors influencing the development of effective WN virus-specific CD4 T cell responses, specifically the sites in virus proteins giving rise to dominant CD4 T cell epitopes, remain unknown.

**Figure 1:**
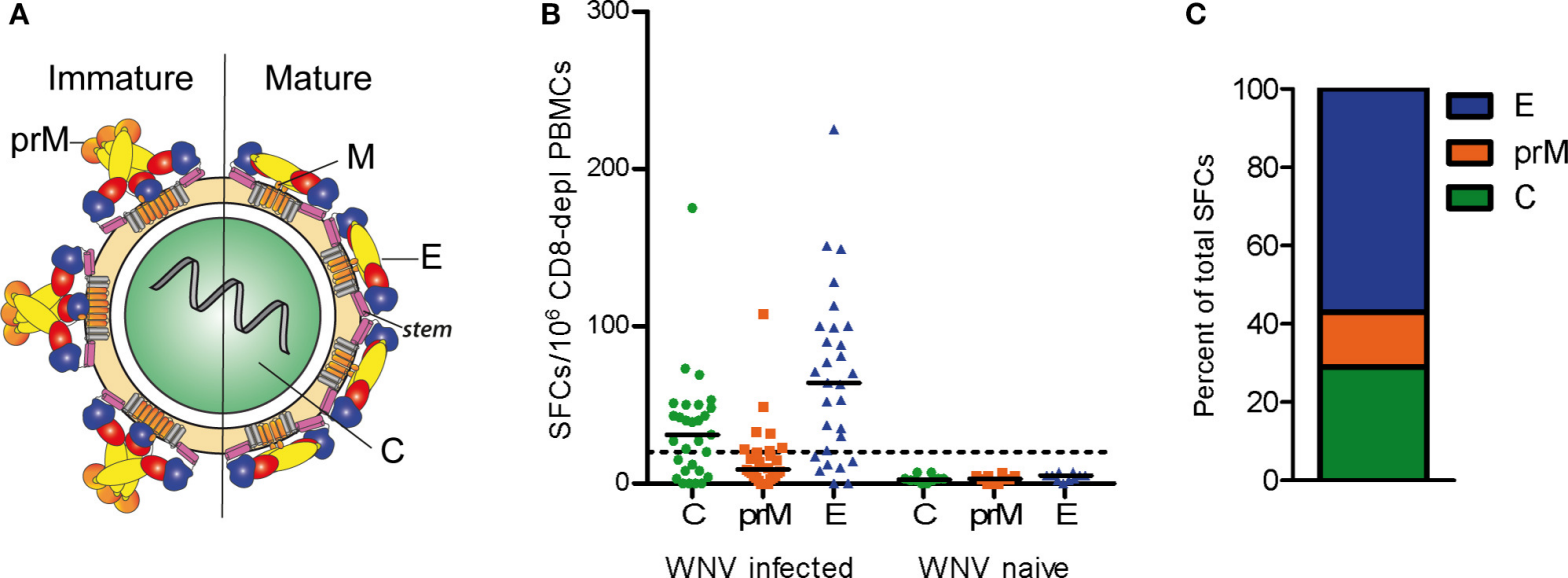
CD4 T Cell Responses to WN Virus. **(A)** Schematic representation of immature and mature flavivirus particles. The virion contains three structural proteins: C (capsid), prM (pre-membrane) in immature and M (membrane) in mature virus particles, and E (envelope). The E protein consists of three domains, DI (red), DII (yellow), DIII (blue), as well as the stem (purple) and transmembrane region (gray). **(B)** Individual CD4 T cell responses from WN virus-infected subjects and naïve controls to WN virus peptide pools covering the entire sequences of structural proteins C, prM and E, as determined by IL-2 ELISPOT assays. Results are shown as spot forming cells (SFCs) per million CD8-depleted PBMCs. Medians are displayed as black lines. The dashed line represents the cut-off value for assay positivity. **(C)** Percentage of spots contributed by C, prM and E in WN virus-infected subjects.

Like flaviviruses in general, mature WN virus particles consist of a nucleocapsid, which is composed of the single-stranded RNA genome and multiple copies of the capsid (C) protein, as well as a lipid envelope with two membrane-associated proteins, the membrane (M) protein, and the envelope (E) protein (Figure 1A). The M protein is generated by proteolytic cleavage of a precursor (prM) that is a component of immature virions (11) (Figure 1A). The E protein contains three distinct domains (domains I–III) and a so-called stem region, followed by two transmembrane domains. Upon entry into host cells, the acidic pH of the endosome triggers an irreversible conformational change in the E protein that leads to membrane fusion and converts the metastable pre-fusion dimer into a stable post-fusion trimer (12, 13). Due to its important cell entry functions, the E protein is the major target of neutralizing antibodies (6–8). CD4 T cells provide essential help for potent neutralizing antibody production by direct cell surface interactions with B cells and the recognition of antigenic peptides presented with major histocompatibility complex class II (MHC II) molecules. Peptides for such direct help can derive from the E protein, recognized by the B cells or the two other structural proteins (M and C) that are co-internalized as part of the virus particle.

The mechanisms influencing peptide presentation are incompletely understood, but a number of factors have been shown to affect the selection of epitopes during different stages of antigen processing. These include protein sequence-related factors, such as protease cleavage sites and MHC binding affinity (14). Furthermore, growing evidence suggests that protein structural features can modulate the efficacy of epitope generation during antigen processing (15–19). In addition, it has been suggested that conformational changes of viral envelope proteins at the early endosomal pH can influence the processing and presentation of certain peptides (20).

An analysis of CD4 T cell epitopes in the context of the atomic structures of viral protein antigens may therefore provide important clues about how the dominance of certain epitopes is controlled by structural features. Recent studies examining immunodominant epitopes in the context of three-dimensional protein structures have led to the identification of critical CD4 T cell epitopes in other flaviviruses, including Zika, yellow fever (YF) and tick-borne encephalitis (TBE) viruses (21–24). Crystallographic and cryo-EM studies have shown that the E proteins of flaviviruses are structurally very similar, whereas their sequences differ by up to 60% among distantly related flaviviruses (25–30). This combination of structural conservation and sequence divergence provides a perfectly suitable model for examining the role of protein structural features as opposed to sequence in the selection of peptides and the dominance of certain epitope responses.

Here, we exploited this model to obtain insights into the selection of CD4 T cell epitopes in WN virus infection. By measuring responses in 29 individuals with either West Nile virus disease (WND) or asymptomatic infection, we identified 67 peptides in the viral capsid and envelope proteins that were recognized in the context of multiple different HLA II alleles. Although WND patients and asymptomatic subjects displayed differences with respect to the recognition of certain epitopes associated with specific HLA alleles, the overall epitope patterns between these groups were similar. Both in C and E proteins, immunodominant epitopes clustered in structurally conserved regions that have also been identified as frequent targets in other mosquito-borne flaviviruses. These data are consistent with an important influence of conserved conformational features in the structural proteins of flaviviruses that shape the specificities and immunodominance of CD4 T cell responses.

## Materials and Methods

### Study Population

A total of 29 WN virus-infected subjects were enrolled in this study (Table 1). The cohort consisted of 11 WND patients (age range, 27–65 years; median age, 51 years; 4 female and 7 male) with moderate to severe symptoms (Table 1), as well as 18 asymptomatic blood donors (age range, 34–70 years; median age, 55 years; 3 female and 15 male) who were recruited through routine screening of blood donations performed by the Austrian Red Cross and the Regional Reference Laboratory of the Lombardy Region (Molecular Virology Unit, Fondazione IRCCS Policlinico San Matteo Pavia) in northern Italy.

**Table 1:** Characteristics of West Nile virus-infected subjects.

		**Diagnosis of WNV infection**		**IL-2 ELISPOT result**		
**Patient**	**Symptoms**	**WNV RNA^a^**	**WNV** **IgM**	**E**	**prM**	**C**
501	Asymptomatic	pos	Pos	37	<20	40
502	Asymptomatic	pos	Pos	21	<20	<20
503	Asymptomatic	pos	Pos	113	33	48
504	Asymptomatic	pos	Pos	35	<20	<20
505	Asymptomatic	pos	Pos	77	22	43
506	Asymptomatic	pos	Pos	70	<20	20
507	Fever, rash, muscle aches	pos	Pos	<20	<20	<20
508	Asymptomatic	pos	Pos	<20	<20	<20
509	Asymptomatic	pos	Pos	99	<20	22
510	Fever, fatigue, muscle aches	pos	Pos	64	<20	31
511	Asymptomatic	neg	Pos	<20	<20	<20
512	Fever, rash, headache	neg	Pos	88	<20	50
513	Fever, headache, rash	pos	Pos	71	<20	53
514	Asymptomatic	pos	Pos	52	<20	27
515	Asymptomatic	pos	Pos	81	21	42
516	Asymptomatic	pos	Pos	53	23	<20
517	Fever, rash	pos	Pos	30	<20	27
518	Fever, rash, ocular pain	pos	Pos	100	<20	50
519	Fever, encephalitis	pos	Pos	225	108	175
520	Asymptomatic	neg	Pos	<20	<20	<20
521	Asymptomatic	pos	Pos	151	32	51
522	Asymptomatic	pos	Pos	149	<20	39
523	Fever, rash	pos	Pos	63	<20	43
524	Fever, rash, muscle aches, ocular pain	pos	Pos	90	<20	40
525	Fever, rash, fatigue, vertigo, nystagmus	pos	Pos	100	20	69
526	Fever, rash, muscle aches, fatigue	pos	Pos	128	49	73
701	Asymptomatic (fatigue)	neg	Pos	<20	<20	<20
705	Asymptomatic	pos	Pos	<20	<20	<20
712	Asymptomatic	pos	Pos	<20	<20	<20

a *WN virus PCR performed in plasma, whole blood, serum and/or cerebral spinal fluid*.

At the time of diagnosis, 25 out of 29 (86%) individuals had detectable WN virus RNA in blood samples, and 29 out of 29 (100%) had detectable WN virus IgM (Table 1). WN virus infection was confirmed in all study participants by neutralization tests, as described below. Previous infection with mosquito-borne flaviviruses from other serocomplexes was analyzed by neutralization assays, as described in detail below. At the time of diagnosis, all 29 participants were negative in Zika neutralization tests. In addition, previous DEN virus infection was ruled out in 28 out of 29 subjects using DEN immunoassays after depletion of broadly flavivirus cross-reactive antibodies, as described below. As a control, samples obtained from 10 flavivirus-naïve individuals were analyzed.

### Ethics Statement

The study was performed in accordance with the recommendations of the Declaration of Helsinki, and all study participants provided written informed consent. The ethics committee of the Medical University of Vienna, Austria, approved the study protocol (approval no. 1295/2016). Patient samples were processed in accordance with biosecurity regulations and institutional safety procedures.

### Detection of West Nile Virus RNA

WN virus PCR was performed as described previously (31). Briefly, WN virus RNA was extracted from plasma, whole blood, serum and/or cerebral spinal fluid using NucliSENS easyMAG extractor (BioMérieux, Marcy l'Etoile, France). For the detection of WN virus lineages 1 and 2, a real-time TaqMan PCR with primers and probe located within the conserved WN virus 3′-noncoding region was used (32).

### Detection of West Nile Virus IgM

WN virus-specific IgM antibodies were detected in a capture ELISA using purified formalin-inactivated WN virus (31) and a WN virus-specific monoclonal antibody E16 for detection (33).

### Neutralization Tests

Neutralization tests (NTs) to confirm WN virus infection were performed with follow-up samples from all individuals, as described previously (31). Briefly, duplicates of serial 2-fold dilutions of heat-inactivated serum samples were incubated with 30–60 TCID_50_ (50% tissue culture infective dose) of WN virus strain NY99 for 1 h at 37°C. Vero cells (ECACC) were added and incubation was continued for 4–6 days at 37°C. The presence of infectious (non-neutralized) virus was assessed by microscopic evaluation of cytopathic effects. Virus NT titers were expressed as the reciprocal of the serum dilution required for hundred percent protection against virus-induced cytopathic effects. Virus NT titers ≥20 were considered positive. Zika virus-specific neutralizing antibodies were determined in Vero cells (23). Duplicates of serial 2-fold dilutions of heat-inactivated serum samples were incubated with 30–60 TCID_50_ Zika virus (strain H/PF 2013) for 1 h at 37°C. Vero cells were added and incubation was continued for 3–4 days. Virus NT titers were expressed as the reciprocal of the serum dilution required for hundred percent protection against virus-induced cytopathic effects. Virus NT titers ≥20 were considered positive (23). YF and TBE NTs were carried out in baby hamster kidney cells (BHK-21, ATCC) (23, 34). Duplicates of serial 2-fold dilutions of heat-inactivated serum samples were incubated with YF-17D virus or TBE virus strain Neudoerfl for 1 h at 37°C. BHK cells were added and incubation was continued for 3–4 days. YF NT titers were expressed as the reciprocal of the serum dilution required for hundred percent protection against virus-induced cytopathic effects (23). Virus NT titers ≥20 were considered positive. In the case of TBE, the presence of virus was measured in the supernatants using ELISA. Neutralization titers were defined as the reciprocal of the plasma dilution that gave a 90% reduction in the absorbance readout in the assay compared to the control without antibody (23). Virus NT titers ≥10 were considered positive. Positive NT titers for YF or TBE or both were obtained in 0 (0%), 21 (72%), and 3 (10%) cases, respectively. Flavivirus-naïve control samples were negative in neutralization assays. Neutralization assays using infectious virus were performed under biosafety level BSL3.

### Antibody Depletion

Depletion of cross-reactive antibodies and dengue IgG ELISAs (Euroimmun) were performed to rule out previous DEN virus infection (23). Broadly cross-reactive antibodies were depleted from serum samples using a strep-tagged soluble E protein (sE, lacking the stem-anchor region) from Rio Bravo virus (35), coupled to StrepTactin XT spin columns (IBA GmbH, Göttingen, Germany). Residual DEN-IgG reactivity was below the detection limit in 28 of 29 patients, indicating that they were DEN-naïve. In the depleted serum of one donor (#705) DEN-IgG Abs were not removed by Rio Bravo virus E protein.

### Preparation of Blood Samples

Peripheral blood mononuclear cells (PBMCs) were separated from whole-blood samples using Ficoll-Paque Plus™ (GE Healthcare) and were cryopreserved in liquid nitrogen, as previously described (23). PBMCs were thawed and depleted of CD8-positive cells using magnetic beads coupled with anti-CD8 antibody and LD columns (Miltenyi Biotec GmbH, Germany), as previously described (24). The depleted PBMCs were incubated overnight in serum-free medium (AIM-V; Gibco) at 37°C in 5% CO_2._ For use in ELISPOT assays, cells were resuspended at a final concentration of 2 × 10^6^ cells/ ml in AIM-V. The purity and viability of CD8-depleted PBMCs in each sample was assessed using anti-CD8-APC, anti-CD3-PE, anti-CD4-PacificBlue™, and 7-aminoactinomycin D (BD Bioscience) and flow cytometry (23). After depletion, CD8-positive cells were <1%. Plasma and serum samples were stored at −20°C.

### Peptides

Peptides were purchased from JPT Peptide Technologies (Berlin, Germany). Peptide purity was >70%. Peptides were quantified and verified for correct sequence by JPT, using high-performance liquid chromatography. The average purity was >85%. Lyophilized peptides were dissolved in dimethyl sulfoxide and diluted in AIM-V medium (23). A total of 190 peptides (15 mer) overlapping by 11 amino acids which cover the entire sequences of C, prM, and E from the WN virus strain Austria/2008 (protein accession code: AGX89731). Three master pools covered the C, prM, or E protein sequences. Matrix pools of C (*n* = 10) and E (*n* = 22) contained up to 14 peptides with each peptide present in two distinct pools. All positive results obtained with the matrix pools were confirmed by testing the samples with single peptides in independent experiments.

### IL-2 ELISPOT Assay

IL-2 ELISPOT assays were performed as previously described (22–24). Briefly, plates (Merck-Millipore) were coated with 1 μg anti-IL-2 antibody (3445-3-1000, Mabtech). For blocking, RPMI 1640 medium (Sigma) containing 10% human serum, 1% penicillin/streptomycin/glutamine (Gibco), and 1% non-essential amino acids (Sigma) was used. CD8-depleted PBMCs (2 × 10^5^/ per well) were incubated at 37°C and 5% CO_2_ for about 45 h with peptides (final peptide concentration 2 μg/ml), AIM-V medium (negative control) or phytohemagglutinin (PHA, Sigma) (final concentration 0.5 μg/ ml, positive control). After washing, spots were developed with 0.05 μg biotin-conjugated anti-IL-2 antibody (3445-6-250, Mabtech), streptavidin-coupled alkaline phosphatase (ALP; 1:1000, 3310-10, Mabtech), and 5-bromo-4-chloro-3-indolylphosphate/nitroblue tetrazolium (BCIP/NBT; B5655, Sigma). The plates were analyzed using a Bio-Sys Bioreader 5000 Pro-S/BR177 and Bioreader software generation 10. Data were calculated as spot forming cells (SFCs) per 10^6^ CD8-depleted PBMCs after subtraction of the spots from the negative control (mean spot number from three to four unstimulated wells). The response to a single peptide was defined positive if the corresponding master pool, matrix pool as well as single-peptide testing yielded >20 SFCs per 10^6^ CD8-depleted PBMCs (22, 23).

### Structural Analysis and Comparison of Flavivirus Epitopes

Experimentally identified WN virus epitopes were assigned to the crystallographic or cryo-EM structures of the WN virus sE protein monomer (PDB 2I69) (25), the Kunjin virus (KUNV) C protein (PDB 1SFK) (36), as well as the Japanese encephalitis (JE) virus E dimers (PDB 3P54 and 5WSN) (37, 38) and the St. Louis encephalitis (SLE) virus E trimer (PDB 4FG0) (39), all belonging to the same serocomplex, using PyMol (Schrödinger LLC, https://pymol.org/). For comparisons, all epitopes were derived from *ex vivo* ELISPOT assays obtained with human CD4 T cells and peptides that span the entire protein sequences (22, 23, 40, 41). Crystallographic structures used for assignment of experimentally identified epitopes were KUN virus C (PDB 1SFK), Zika sE (5LBV) (26), DEN-2 sE (PDB 1OAN) (27), and YF sE (PDB 6EPK) (28). For comparison of all mosquito-borne flavivirus protein sequences and epitopes of C and E, multiple sequence alignments were performed (GenBank: Zika virus KJ776791; DEN 1–4 viruses AF226687, M29095, DQ863638, GQ398256; YF virus CAA27332; WN virus DQ211652 and JE virus D90194) using Clustal Omega and manually refined, as described previously (42–44). This analysis also included predicted MHC II-binding peptides derived from DEN virus C or E that were positive in at least two responders (45, 46), as well as epitopes from the JE virus C and E proteins (47).

Structural similarity of the identified epitope regions was assessed using crystallographic structures of the WN virus (PDB 2I69), YF virus (PDB 6EPK), Zika virus (PDB 5LBV), and DEN virus sE proteins (PDB 1OAN) lacking the stem-anchor region. For selected WN virus epitope regions E41, E149, E245, and E381, as well as for the corresponding regions in YF, Zika, and DEN viruses, structural superposition of WN virus sE domains I (E41, E149), II (E245), or III (E381), respectively onto those of YF, Zika and DEN viruses was performed using the PyMOL algorithms “align” and “super” (48, 49). Root-mean-square deviation (RMSD) of selected epitope regions was computed through the PyMOL “rms_cur” command (without further fitting).

### HLA Genotyping

HLA-DRB1, HLA-DQA1, HLA-DQB1, HLA-DPA1, and HLA-DPB1 alleles from all study participants were genotyped by sequencing of the whole gene. Genotyping of HLA-DRB3/4/5 allele genotyping was done from exon 2 to the 3′UTR by next generation sequencing as described previously (22, 50). Briefly, long-range PCR products were generated by allele-specific primers and libraries generated by enzymatic fragmentation of the amplicons. After ligation to barcoded adapters the libraries were subjected to an emulsion PCR and the product sequenced on an Ion Torrent sequencing device (IonTorrent PGM, Thermo Fisher Scientific Inc., Waltham, MA). Sequences were analyzed using HLATypeStream (Thermo Fisher Scientific Inc., Waltham, MA) and NGS GenDX (GenDX, Utrecht, NL) software. HLA genotypes were assigned on basis of the IMGT/HLA database. For HLA-DQ predictions, haplotype compositions of –DQA1 and –DQB1 were assigned, based on the strongest linkage between these alleles, according to the reference HLA haplotype frequency standards (51).

### *In silico* Prediction of MHC II Binding

Predictions were done in July 2019 using the Immune Epitope Database (IEDB) analysis tools “IEDB recommended” (www.iedb.org) and “NetMHCIIpan” (52, 53). C and E protein sequences of WN virus (protein accession code: AGX89731) were entered separately. Predictions were done for all experimentally tested peptides and the HLA class II alleles from all individuals (Table S2). For individual predictions, query submission was automated using the framework provided by the PeptX project (54).

### Data Analysis

Statistical analyses were performed with GraphPad Prism, version 5. Comparisons of overall CD4 T cell reactivity in asymptomatic donors and WND patients was done using a non-parametric Kruskal-Wallis test. A Wilcoxon-signed rank test was used for comparison of the number of peptides that induced a response in asymptomatic donors and WND patients. A Spearman correlation coefficient was calculated for evaluation of two-by-two correlations. The strength of associations between expression of a specific allele and detection of a positive epitope response was calculated as described previously (55, 56). Briefly, odds ratios (ORs) were calculated, according to the following formula: *OR* = $\frac{\left(A+R+\right)x\left(A-R-\right)}{\left(A-R+\right)x\left(A+R-\right)}$.

*A*+*R*+ = number of subjects who expressed the specific allele and had a positive response to the specific peptide, *A*+*R*− = number of subjects who expressed the specific allele but did not have a positive response to the specific peptide, *A*−*R*+ = number of subjects who did not express the specific allele but had a positive response to the specific peptide, *A*−*R*− = number of subjects who did not express the specific allele and did not have a positive response to the specific peptide (56). Fisher's exact test was applied to calculate the statistical significance of the association between one or more specific HLA alleles and the response to a specific peptide (55). A *p*-value < 0.05 was considered statistically significant.

## Results

### CD4 T Cell Response Against WN Virus Structural Proteins

We first investigated the overall reactivity of CD4 T cell responses to peptides derived from the WN virus structural proteins, the capsid C, membrane prM/M and envelope E (Figure 1A). PBMC samples from WN virus-infected individuals were depleted of CD8-positive cells and tested in IL-2 ELISPOT assays with pools of overlapping 15 mer peptides that cover the entire sequence of each of the structural proteins. The study population consisted of 29 subjects (18 asymptomatic/11 WND patients; age range 27–70 years) (Table 1), recruited at a mean of 40 ± 6 days after diagnosis. In terms of the overall magnitude, highest responses were elicited by E peptides, which accounted for 57% of the total response, followed by C and prM peptides, which accounted for 29 and 14% of the total response, respectively (Figures 1B,C). In contrast, none of the flavivirus-naïve individuals showed a response to any of the C, prM and E peptides, confirming the specificity of this assay (Figure 1B).

**Figure 2:**
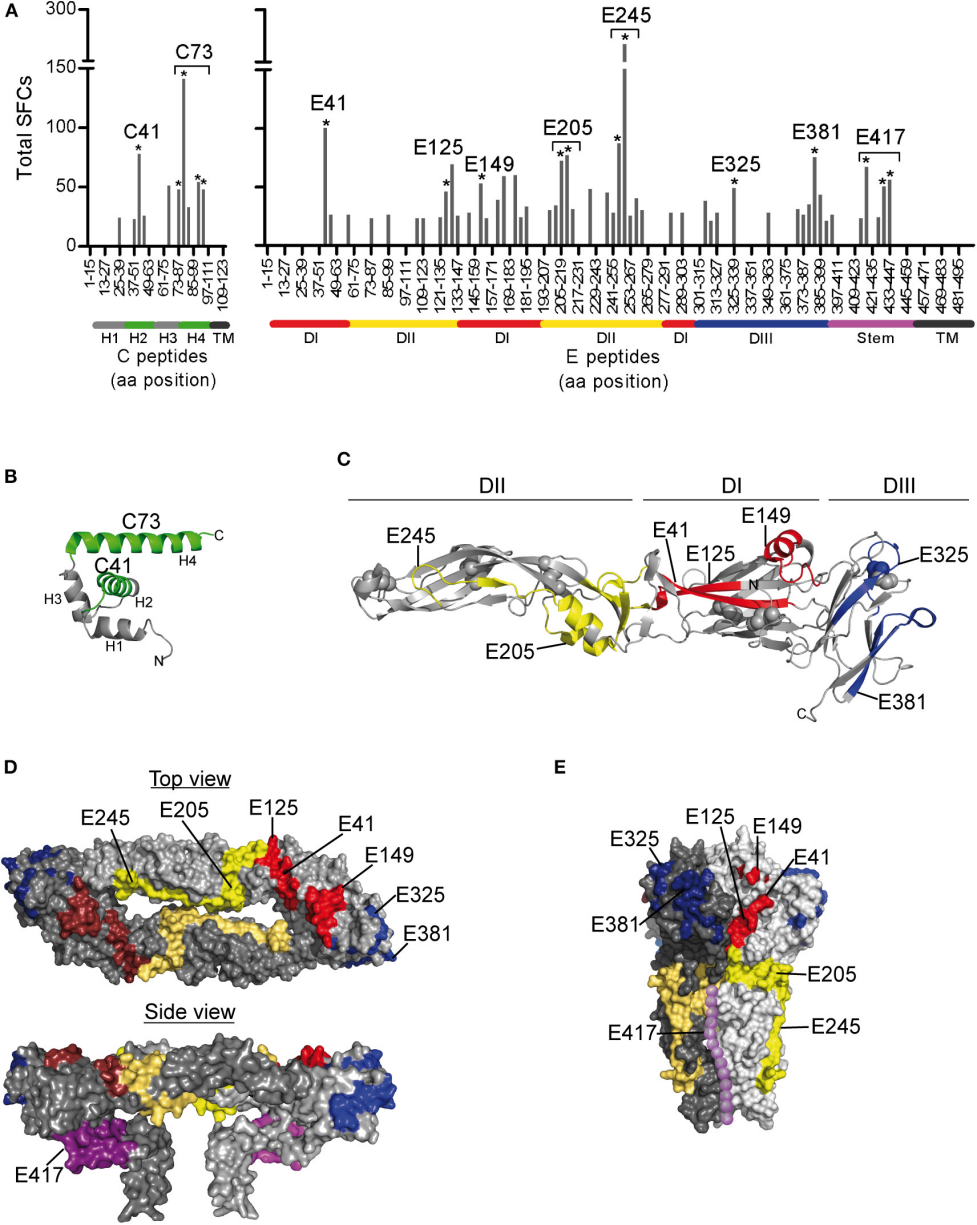
Mapping of CD4 T Cell Epitopes to WN Virus C and E Proteins. **(A)** Total spot counts of positive responses to single peptides. Amino acid positions of peptides in C and E protein sequences are indicated on the x-axes. Peptides recognized in two or more individuals are indicated by asterisks and are designated by the first amino acid of the 15 mer peptide. **(B)** Ribbon diagram of the WN virus lineage 1 variant Kunjin C protein (PDB 1SFK) (36), comprising four helices (H1-H4). **(C)** Ribbon representation of the WN virus soluble E (sE) ectodomain (PDB 2I69; side view) (25), consisting of three domains (DI-DIII). **(D)** Surface representation of the Japanese encephalitis virus pre-fusion sE dimer (top view, PDB 3P54) (37) and the full-length E dimer (side view, PDB 5WSN) (38). **(E)** Surface depiction of the St. Louis encephalitis virus post-fusion E protein trimer (PDB 4FG0; side view) (39); the unresolved stem is added schematically to the structure and shown as dotted line along domain II (purple). Immunodominant WN virus peptides are colored as follows: C, green; E, DI-red, DII-yellow, DIII-blue, and stem-purple.

To determine the epitope specificities of responses, we performed IL-2 ELISPOT assays using peptide matrix pools and single peptides covering the entire C and E proteins. As observed for other flaviviruses (22–24), the prM peptides yielded only weak positive signals (Figure 1B), and therefore single peptide analysis was not performed. On average, individual responses were directed against five CD4 T cell epitopes accounting for a median 148 spot forming cells (SFCs) (range, 31–414 SFCs). There were no significant differences in the breadth or magnitude of responses between WND patients (median, 6.0 epitopes; 205 SFCs, range: 88–414) and asymptomatic subjects (median, 3.5 epitopes, 94.5 SFCs, range: 31–412). The cumulative number of spots for each peptide, using the results from all subjects obtained with the single-peptide testing, is displayed in Figure 2A. Individuals with asymptomatic infection and WND yielded similar epitope response patterns (*p* < 0.0001, Spearman correlation *r* = 2.8) (Figure S1). Overall, we identified 61 peptides that induced a CD4 T cell response, indicating a substantial heterogeneity in the individual epitope reactivities (Table S1). However, closer inspection of the data showed that few epitopes accounted for the majority of the response. These epitopes, recognized in two or more individuals, accounted for 50% of the total SFC responses and were defined as immunodominant. The immunodominant peptides clustered in two antigenic regions of C (C41 and C73) and in eight regions of E (E41, E125, E149, E205, E245, E325, E381, and E417) (Figure 2A).

### Structural Analysis of Immunodominant Sites in C and E Proteins

Since the structural context of an epitope has been proposed to influence immunodominance (15–19, 57, 58), we next analyzed the structural characteristics of the protein regions from which the identified epitopes were derived. For this purpose, we mapped the immunodominant epitopes on the three-dimensional structures of the WN virus C and E proteins. As illustrated in Figure 2B, responses to the C protein were mainly focused to helices H2 and H4, whereas helices H1, H3, and the N-terminal part of C did not elicit dominant responses. In the E protein (Figure 2C), immunodominant epitopes clustered in each of the three domains [three in domain I (E41, E125, and E149), two in domain II (E205 and E245), two in domain III (E325, E381)] and in the stem region (E417). As shown in Figures 2D,E, epitopes from all three domains were located at the outside of the E protein in its dimeric conformation as well as in its trimeric form. In addition, the trimer exposes an epitope (E417) in the stem region (Figure 2E), whereas the stem is hidden below the E ectodomain in its pre-fusion conformation (Figures 1A, 2D).

**Figure 3:**
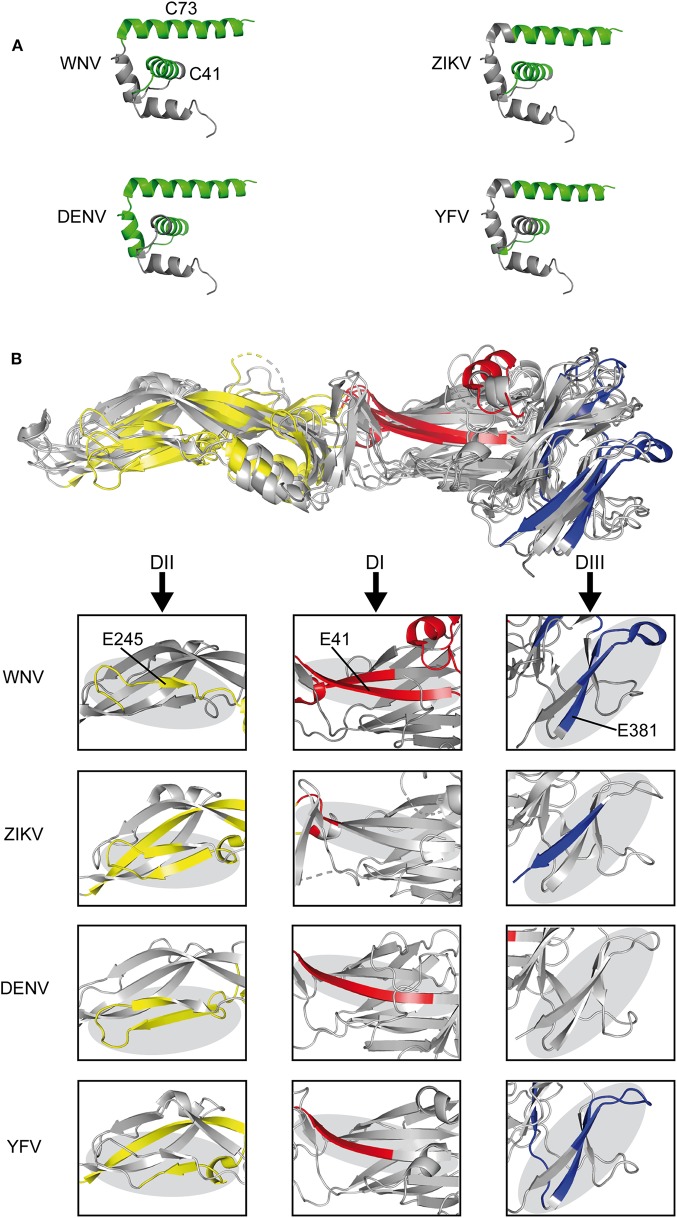
Comparison of CD4 T Cell Epitope Sites in C and E Proteins of Mosquito-borne Flaviviruses. **(A)** Ribbon representation of the flavivirus Kunjin C protein (PDB 1SFK) (36) highlighting epitopes recognized in WN, Zika (23), DEN (40, 41), and YF viruses (22). **(B)** Envelope protein structure alignment of WN (PDB 2I69) (25), Zika (PDB 5LBV) (26), DEN (PDB 1OAN) (27), and YF (PDB 6EPK) (28) viruses with assigned epitopes. Boxes highlight immunodominant epitope regions in each of the viruses. Immunodominant epitopes are colored as follows: C, green; DI-red, DII-yellow, and DIII-blue.

We next compared the experimentally identified WN virus epitopes with the epitopes previously determined for other mosquito-borne flaviviruses, including YF, Zika, and DEN viruses. All of these epitopes were identified in *ex vivo* ELISPOT testing of human CD4 T cells and overlapping peptides covering the entire protein sequences (22, 23, 40, 41). As can be seen in Figure 3A, immunodominant regions in C were uniformly located at positions in helices H2 and H4 (C41, C73). In E (Figure 3B), three of the dominant WN virus epitopes (E41, E245, and E381) mapped to sites harboring immunodominant epitopes in all mosquito-borne flaviviruses, except for E381, which was not identified in DEN virus infections. Structurally, these common epitope regions encompassed ß-sheets as well as loops that are about 20 amino acids long and are devoid of disulphide bridges (Figure 3B). In addition, we identified two dominant sites (E125 and E205) that were congruent between WN, Zika, and DEN viruses. These data together with all epitopes identified by *ex vivo* ELISPOT assays are shown in Figure S2. Two WN virus epitopes (E325 and E417) were not present in DEN, Zika, and YF viruses, but overlapped with those previously identified in Japanese encephalitis virus, a closely related flavivirus of the same serocomplex (Figure S2). In contrast, one epitope, E149, which involves an extra α-helix in DI, was only found in WN virus (Figure S2).

**Figure 4:**
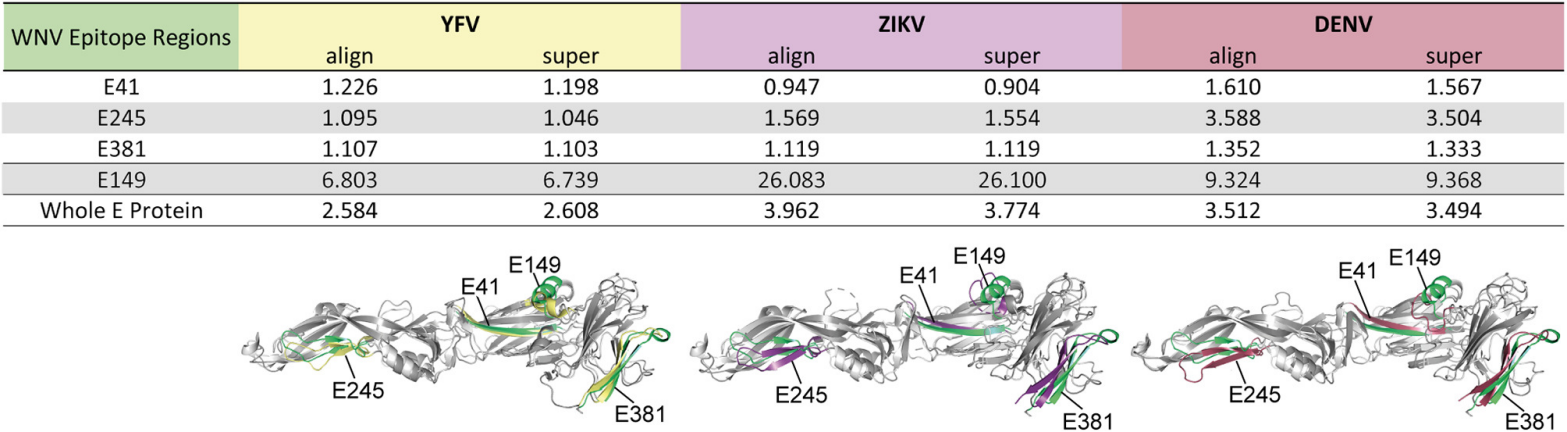
Structural Similarity of Common Flavivirus Epitope Regions in E. Pairwise structural alignments for E proteins between WN virus (PDB 2I69) (25) and YF (PDB 6EPK) (28), Zika (PDB 5LBV) (26), or DEN (PDB 1OAN) (27) viruses. Structural elements encompassing the selected epitopes were analyzed, using the PyMOL algorithms “align” and “super.” These elements are colored in green (WN virus), yellow (YF virus), purple (Zika virus), and red (DEN virus). RMSD values are given in Ångström.

As a further estimate for the structural homology of the common flavivirus epitope sites, we computed the RMSD of selected epitope regions after pairwise structural alignments of WN virus sE protein domains with those of YF, Zika and DEN viruses, respectively. The analysis included epitope regions E41, E245, and E381, which were common among flaviviruses (Figure 3B), as well as epitope E149, which had no equivalent in other flaviviruses (Figure S2). As can be seen in Figure 4, alignment of sE proteins yielded RMSD values of 2.58–3.96Å, consistent with those for homologous proteins (48, 59). The ß-sheet encompassing E41 yielded very low RMSD values (0.90–1.61 Å), and values below 2 Å were also observed for all pairwise comparisons of E245 and E381, except for E245 in DEN virus, which had a value of 3.50 to 3.59 Å. In contrast, E149 yielded RMSD values of 6.74 to 26.10 Å, indicating a structurally divergent region. In fact, in WN virus, this site harbors an extra α-helix protruding from the upper part of E DI, which is absent in Zika and DEN viruses (Figure 4). It is notable that an α-helix occurs at a similar sequence position in the YF virus E protein, however this helix exhibits a ~90° twisted orientation and sticks closer to the surface of E DI (Figure 4). Together, the data suggest that the common epitope sites resulted from conserved structural features that favor their selection across different flaviviruses.

### HLA II Binding Prediction and Association of Epitope Responses

**Figure 5:**
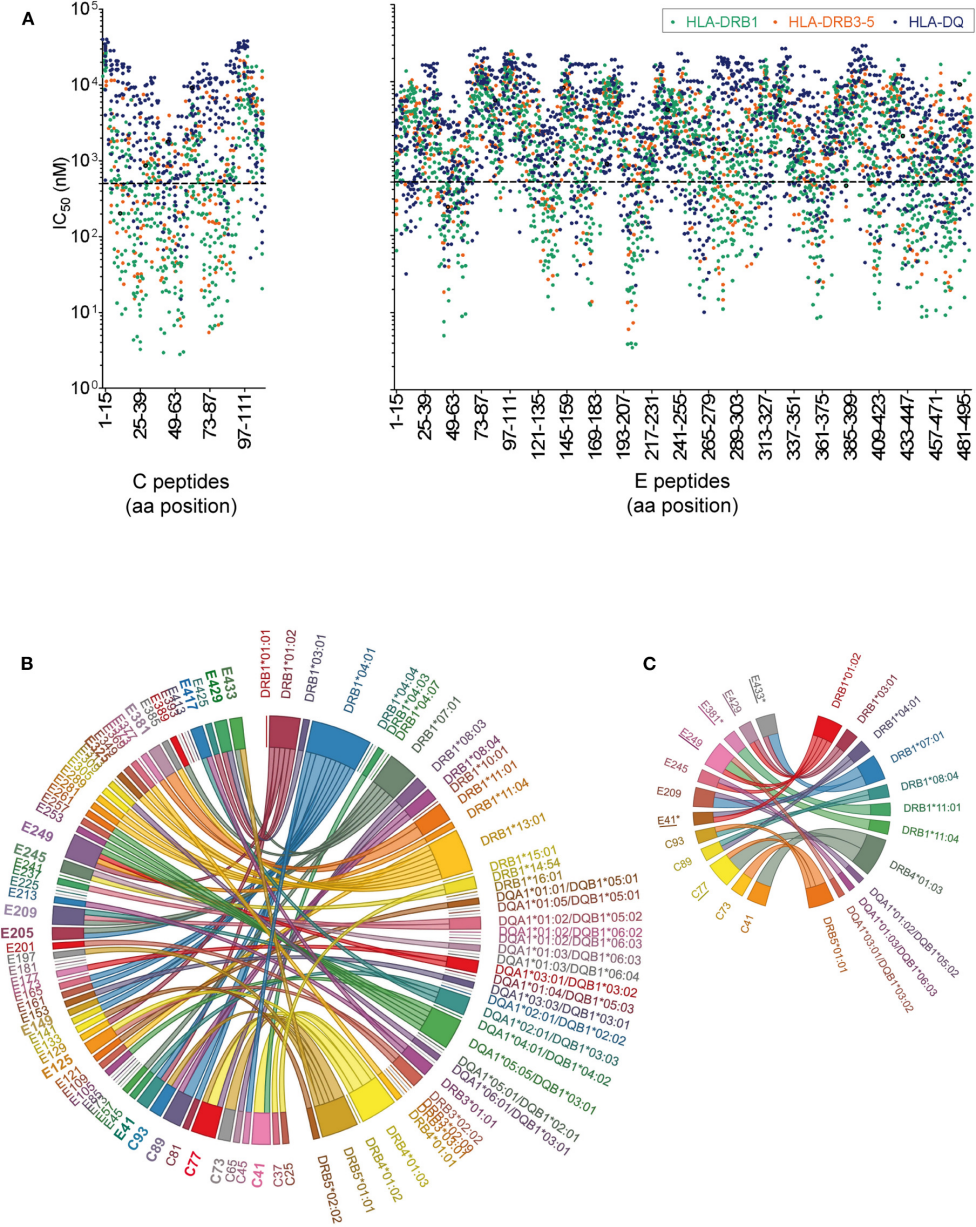
Peptide-MHC II Binding Prediction and HLA Association of West Nile Virus Epitopes. **(A)** Predicted IC_50_ values for experimentally tested peptides and the HLA II alleles of the study cohort. Peptides with the highest affinities locate toward the bottom of the y-axes and have IC_50_ values below 500 nM (dotted line). **(B)** The chord diagram demonstrates for each individual, the experimentally identified epitope on the left side of the circle, connected with the HLA II allele predicted with best percentile rank on the right side of the circle. Immunodominant peptides are in bold. **(C)** Chord diagram indicating HLA II alleles associated with immunodominant epitopes in C and E. Epitopes detected only in asymptomatic individuals are indicated by asterisks. Epitope sequences previously identified by HLA II tetramer staining (60) are underlined.

The immunogenicity of CD4 T cell epitopes is also determined by the affinity of a particular peptide to an individual's HLA repertoire. As a prerequisite for MHC II affinity prediction, HLA genotyping of class II alleles (DRB1, DRB3, DRB4, DRB5, DQA1, DQB1, DPA1, and DPB1) was performed for each of the 29 donors (Table S2). First, HLA binding affinity was predicted for the entire set of experimentally tested WN virus C and E peptides (*n* = 150) using the NetMHCIIpan algorithm provided by the Immune Epitope Database (IEDB). Approximately 1,400 peptide-MHC II pairs yielded IC_50_ values of < 500 nM, indicating likely binders (52, 53). Analysis of the data displayed in Figure 5A revealed that 97% of the peptides were predicted with several different HLA alleles (up to 30 different alleles/peptide), and only 3% were predicted exclusively for one or no allele. We therefore conclude that the majority of peptides derived from C and E proteins could theoretically be presented by a large number of different MHC molecules expressed in this study cohort. However, a considerable number of frequently predicted peptides did not elicit a dominant CD4 T cell response in ELISPOT assays. This was especially the case for peptides derived from the transmembrane anchors (aa 449-501), as well as for peptides from domains II (aa 261-295) and III (aa 349-375) (Figure 5A). These findings are consistent with the notion that HLA binding is a necessary but not sufficient requirement for T cell responses (61, 62).

In addition to this population-based approach, we assessed specifically for each individual which of the HLA alleles had the best prediction value (best percentile rank) for the peptides detected in ELISPOT assays, using the Immune Epitope Database (IEDB) analysis tool “IEDB recommended” (www.iedb.org). In this analysis, 82% of all experimentally identified peptides had an IEDB percentile rank of ≤20 for the individual HLA II alleles. For the illustration of the results in Figure 5B, a chord diagram was used which presents all experimentally identified epitopes on the left side of the circle, connected to the HLA II alleles predicted with best percentile ranks on the right side of the circle. Notably, more than 50% of the immunodominant peptides were predicted to bind quite strongly, with percentile ranks <1.5, and 90% with percentile ranks <10 (Table S3). These data indicate that most of the immunodominant epitopes were predicted as strong binders.

To assess the strength of association between a particular HLA allele and the epitope response in each individual, we calculated odds ratios (ORs) for all combinations of HLA alleles and dominant peptides shown in Figure 5B. In the case of peptides predicted as binders for more than one HLA allele, combined calculations for these alleles were performed, as described previously (55). Evidence for significant associations with specific HLA alleles were obtained for most but not all immunodominant peptides (Table S3 and Figure 5C). The data show that, while only 25% (3/12) of these epitopes were associated with one single HLA allele (C41-DRB4^*^01:03, C73-DRB5^*^01:01 and E381-DRB1^*^11:01), 75% (9/12) were associated with two to four different HLA molecules (Figure 5C). These findings indicate that most immunodominant WN virus epitopes are recognized in the context of different HLA molecules, consistent with the well-known phenomenon of promiscuous binding of immunogenic peptides (63, 64). The data also corroborate previous results obtained with tetramers using different HLA-DR molecules (60) which match almost half of the C and E peptides identified in the present study (peptide matches are underlined in Figure 5C). The OR calculations were also applied to asymptomatic or WND subjects separately. Three of the epitopes yielded a significant HLA association in asymptomatic donors but not in WND patients. These epitopes (E41-IDVKMMNMEAANLAD-HLA-DRB1^*^01:02 and -DRB1^*^04:01 [*p* = 0.039; OR 29.0], E381-DSYIVVGRGEQQINH-HLA-DRB1^*^11:01 [*p* = 0.0196; OR 51.7] and E433-GVFTSVGKAIHQVFG-HLA-DRB1^*^07:01 [*p* = 0.039; OR 29.0]) were detected experimentally only in asymptomatic but in none of the WND subjects and are indicated by asterisks in Figure 5C.

## Discussion

Factors responsible for controlling the immunodominance of certain epitope sites of viral proteins in CD4 T cell responses are poorly defined but can be of great importance for generating protective immunity. A better understanding of these fundamentals requires unbiased exploration of the *ex vivo* T cell epitope repertoire, defining the structural features and the host genetic factors that underlie the preferential selection of epitopes. Toward these objectives, we examined the CD4 T cell epitope specificities in 29 WN virus-infected individuals, using a library of overlapping peptides covering the entire virion proteins. We focused on CD4 T cells directed against epitopes within the virus structural proteins, because these can mediate help to B cells by direct cell-cell interactions and are therefore essential for the production of neutralizing antibodies, the major protective correlate in flavivirus infections (6–8).

Our study demonstrates that dominant CD4 T helper cell responses are directed to two helices in the virus capsid and to surface-exposed regions of the viral envelope protein E in its pre- and post-fusion forms. This immunodominance pattern was strikingly similar to those previously identified for distantly related flaviviruses, including Zika (23), DEN (40, 41), and YF (22) viruses. Especially with epitopes of the capsid protein, the immunodominance patterns were remarkably similar for each of the different flaviviruses. Also in the case of E, we observed a strong overlap of specific epitope regions, particularly with respect to three epitope sites (E41, E245, and E381) that were almost uniformly present in the flaviviruses examined. In our study, epitope reactivities of WN virus-specific CD4 T cells were determined in an *ex vivo* ELISPOT assay using IL-2 that has been applied in a number of previous studies investigating flavivirus-specific CD4 T cell responses (22–24, 65). It was shown that most of the peptide-specific CD4 T cells are polyfunctional (e.g., triple- [IL-2^+^TNF-α^+^IFN-γ^+^] or dual-positive [IL-2^+^IFN-γ^+^]) cells with at least as many CD4 T cells secreting IL-2 as compared to IFN-γ (65). Consistent with these findings, the present analysis revealed similar distributions of epitopes that were identified using IL-2 (22, 23) or IFN-γ ELISPOT assays (40, 41). However, we cannot exclude that our approach could miss epitopes targeted by single IFN-γ positive cells. Further studies that involve dual IFN-γ and IL-2 ELISPOT testing will be required to address this issue.

Analyses in the context of the atomic structures of the corresponding E proteins showed that the common epitopes are displayed in structurally conserved elements and preferentially locate to surface-exposed regions of E (e.g., the domain I-II hinge regions [E41 and E125] as well as the lateral ridge of domain III [E325 and E381]) which have been shown to be important sites for potent neutralizing antibodies against WN virus in humans (33, 66, 67). Our findings are consistent with previous studies demonstrating that CD4 T cell epitopes are preferentially located at exposed protein sites or at flanks of conformationally flexible loops (15–19, 68, 69). Such segments may offer initial cleavage sites for proteolytic processing and are easily accessible binding sites for MHC II molecules (14, 70). Interestingly, the only immunodominant WN virus epitope (E149) that had no overlap with epitopes from other flaviviruses, included an α-helix that is absent from Zika and DEN viruses. Its protrusion from the upper part of DI may increase its accessibility for proteolytic processing and/or MHC binding. Further evidence for the structural influence on epitope selection was obtained by our finding that several peptides predicted to bind a considerable number of HLA molecules were not observed experimentally. This is especially true for peptides from the transmembrane region that are shielded in the lipid bilayer, which might impose strong constraints on peptide generation (24).

In contrast to the other WN virus epitopes identified, the E417 epitope appears to be less accessible in the context of mature virions, because it is sandwiched between the external protein shell and the lipid membrane (Figure 1A). However, upon endocytosis and encounter of the acidic pH in the endosome, flavivirus E proteins undergo a fusogenic conformational change from dimers to trimers, in which the stem is located at the outside (12, 13). Since fusion can already occur at the pH of early endosomes (71), the post-fusion conformation may be an important substrate for generating peptides of the stem region. Such structural aspects may also explain previous observations with TBE virus, which demonstrated that epitopes derived from the stem are immunodominant only in naturally infected patients but not in those vaccinated with a formalin-inactivated vaccine, in which the prefusion conformation of E is stabilized by formaldehyde cross-linking (24). There are many other well-known examples, such as influenza virus or HIV, which undergo conformational changes that may play a role in the preferential selection of certain epitopes. Moreover, studies with influenza virus reported that some peptides are generated only during protein synthesis in infected cells but not upon exogenous uptake of the same antigen (20, 72).

In addition to protein structure-specific factors that are responsible for characteristic immunodominance patterns, our ELISPOT data revealed considerable individual variation in epitope specificities, indicating additional influences of individual-specific factors that control epitope recognition. Evaluations based on *in silico* predicted peptide-MHC II affinities provide evidence that most immunodominant peptides were predicted as strong binders and could potentially be presented in combination with multiple different MHC II molecules. Our results are consistent with recent studies (60), demonstrating that CD4 T cells could be detected in direct *ex vivo* samples by peptide-matched tetramers, which included a number of different alleles. Comparing the characteristics of WN virus-specific CD4 T cells in individuals with either neuroinvasive disease or asymptomatic infection, the same study found that individuals with neuroinvasive disease had higher numbers of virus-specific CD4 T cells (60). This is consistent with the finding that the only patient in our study with neuroinvasive disease (patient #519) demonstrated a substantially higher magnitude of response than other patients, which could be an effect of increased viral replication and/or delayed viral clearance, as described in a significant percentage of patients with WN virus encephalitis (73). Apart from this single case and in line with previous findings (74), we observed no differences with respect to the overall magnitude of response between individuals with WND or asymptomatic infection, and no difference was apparent in the breadth of responses among these groups.

Despite the overall similarities of epitope responses between asymptomatic subjects and WND patients, we did observe certain peptide-HLA combinations which only occurred in asymptomatic individuals. It is unclear at present whether these differences relate to the presence of alleles that protect from WND. An HLA-specific association for both protection and susceptibility to WND was suggested in previous studies (75–77). Additional research is needed to clarify whether such differences could discriminate patients with different disease outcomes. Knowledge of the immunodominant epitopes displayed by such alleles could substantially contribute to our understanding of the determinants that impact the clinical outcome of infection and the efficacy of vaccines.

In summary, we have demonstrated that WN virus infection elicits CD4 T cell responses to peptides that cluster in specific regions of the viral structural proteins and are frequently predicted as strong binders for individual HLA alleles. The immunodominant epitopes locate to structurally conserved antigen regions which are also frequently targeted in other clinically relevant flaviviruses, including yellow fever, dengue and Zika viruses. Together, these data provide novel insights into a key aspect of flavivirus immunity, namely how virion protein structure shapes CD4 T cell responses, which is an important issue to consider in rational vaccine design.

## Data Availability Statement

All datasets generated for this study are included in the article/Supplementary Material.

## Ethics Statement

The studies involving human participants were reviewed and approved by the Ethics committee of the Medical University of Vienna, Austria. The patients/participants provided their written informed consent to participate in this study.

## Author Contributions

MK, JA, FH, SA, KS, and SM performed experiments, reviewed data, and planned experimental strategy. BK, MK, FS, and DF performed bioinformatic analysis. GF and IF performed and coordinated HLA typing. FB, IC, HL, and CJ collected samples and provided clinical information. JA and FH conceived the study. JA wrote the first draft. FH and MK contributed to the writing of the manuscript. All authors critically read and edited the manuscript.

### Conflict of Interest

BK was employed by company Symptoma GmbH, Vienna, Austria. The remaining authors declare that the research was conducted in the absence of any commercial or financial relationships that could be construed as a potential conflict of interest.

## Abbreviations

C: capsid
DEN: Dengue
E: envelope
HLA: human leukocyte antigen
JE: Japanese encephalitis
M: membrane
MHC: major histocompatibility complex
PBMCs: peripheral blood mononuclear cells
prM: pre-membrane
TBE: Tick-borne encephalitis
WN: West Nile
WND: West Nile virus disease
YF: Yellow fever.
