# Supplementary material for: CD4 T Cell Determinants in West Nile Virus Disease and Asymptomatic Infection
Source: Front Immunol. 2020 Jan 23;11:16. doi: 10.3389/fimmu.2020.00016 (PMC6989424; doi:10.3389/fimmu.2020.00016)
Supplement: Supplementary file 1 [file Data_Sheet_1.PDF]

Supplementary Material

CD4 T Cell Determinants in West Nile Virus Disease and Asymptomatic Infection

Maximilian Koblischke[1], Felicia S. Spitzer[1], David M. Florian[1], Stephan W. Aberle[1], Stefan Malafa[1], Ingrid Fae[2], Irene Cassaniti[3], Christof Jungbauer[4], Bernhard Knapp[5], Hermann Laferl[6], Gottfried Fischer[2], Fausto Baldanti[3], Karin Stiasny[1], Franz X. Heinz[1] and Judith H. Aberle[1*]

[1]Center for Virology, Medical University of Vienna, [2]Department of Blood group Serology and Transfusion Medicine, Medical University of Vienna, Vienna, Austria. [3]Molecular Virology Unit, Microbiology and Virology Department, Fondazione IRCCS Policlinico San Matteo; Department of Clinical, Surgical, Diagnostic and Pediatric Sciences, University of Pavia, Italy, [4]Austrian Red Cross, Blood Service for Vienna, Lower Austria and Burgenland, Vienna, Austria, [5]Symptoma, Vienna, Austria; [6]Sozialmedizinisches Zentrum Süd-Kaiser-Franz-Josef-Spital, Vienna, Austria.

***Correspondence:**
Judith Aberle
judith.aberle@meduniwien.ac.at

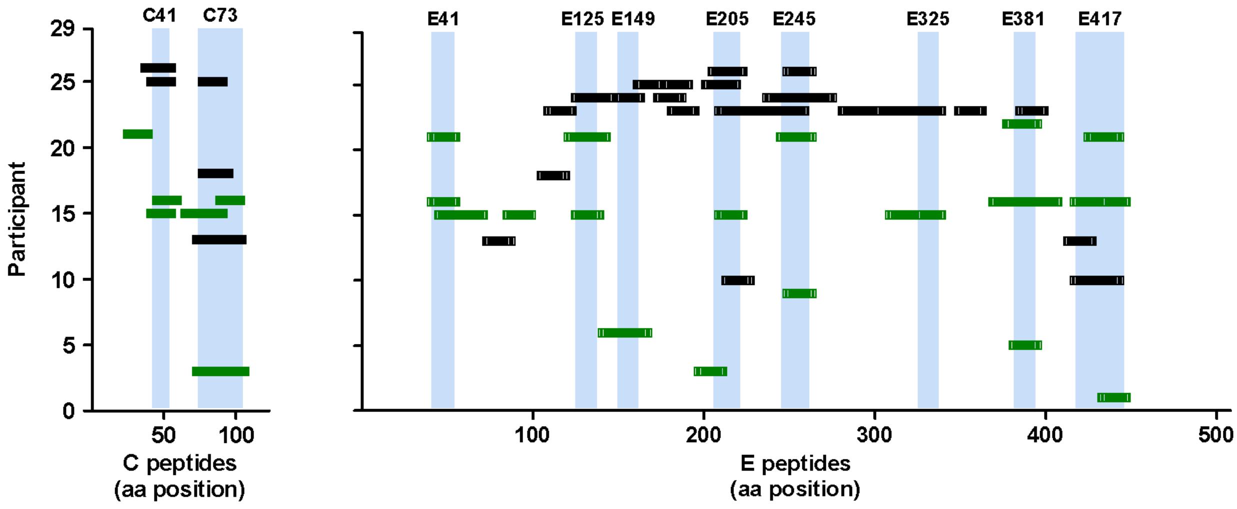

Figure S1. Individual CD4 T Cell Responses. Positive ELISPOT responses to overlapping 15mer peptides from WN virus C and E proteins in 29 WN virus-infected individuals. Each number on the y-axis represents a single subject. Horizontal bars represent responses to single or overlapping peptides from WND patients (black symbols) or asymptomatic subjects (green symbols). Positions of immunodominant epitope regions are indicated by light-blue columns. The x-axis indicates amino acid positions of peptides within C and E proteins.

Table S1. Identified CD4 T Cell Epitopes from West Nile Virus C and E Proteins

Protein	Peptide position[a]	Peptide sequence	Responders [%][b]
C	25-39	LSLIGLKRAMLSL	12,5
	37-51	LIDGKGPIRFVLA	12,5
	41-55	KGPIRFVLALLAF	37,5
	45-59	RFVLALLAFFRFT	12,5
	65-79	VLDRWRGVNKQTA	12,5
	73-87	NKQTAMKHLLSFK	25,0
	77-91	AMKHLLSFKKELG	50,0
	81-95	LLSFKKELGTLTS	12,5
	89-103	GTLTSAINRRSTK	25,0
	93-107	SAINRRSTKQKKR	25,0
E	41-55	IDVKMMNMEAANL	13,3
	45-59	MMNMEAANLADVR	6,7
	57-71	RSYCYLASVSDLS	6,7
	73-87	ACPTMGEAHNEKR	6,7
	85-99	RADPAFVCKQGVV	6,7
	105-119	CGLFGKGSIDTCA	6,7
	109-123	GKGSIDTCAKFAC	6,7
	121-135	CTTKATGWIIQKE	6,7
	125-139	ATGWIIQKENIKY	13,3
	129-143	IIQKENIKYEVAI	6,7
	133-147	ENIKYEVAIFVHG	6,7
	141-155	IFVHGPTTVESHG	6,7
	149-163	VESHGNYSTQIGA	13,3
	153-167	GNYSTQIGATQAG	6,7
	161-175	ATQAGRFSITPSA	6,7
	165-179	GRFSITPSAPSYT	6,7
	173-187	APSYTLKLGEYGE	6,7
	177-191	TLKLGEYGEVTVD	6,7
	181-195	GEYGEVTVDCEPR	6,7
	197-211	DTSAYYVMSVGAK	6,7
	201-215	YYVMSVGAKSFLV	6,7
	205-219	SVGAKSFLVHREW	13,3
	209-223	KSFLVHREWFMDL	20,0
	213-227	VHREWFMDLNLPW	6,7
	225-239	WSSAGSTTWRNRE	6,7
	237-251	ETLMEFEEPHATK	6,7
	241-255	EFEEPHATKQSVV	6,7
	245-259	PHATKQSVVALGS	20,0
	249-263	KQSVVALGSQEGA	26,7
	253-267	VALGSQEGALHQA	6,7
	257-271	SQEGALHQALAGA	6,7
	261-275	ALHQALAGAIPVE	6,7
	281-295	LTSGHLKCRVKME	6,7
	289-303	RVKMEKLQLKGTT	6,7
	305-319	CSKAFKFAGTPAD	6,7
	309-323	FKFAGTPADTGHG	6,7
	313-327	GTPADTGHGTVVL	6,7
	325-339	LELQYTGTDGPCK	13,3
	349-363	LTPVGRLVTVNPF	6,7
	369-383	SKVLIELEPPFGD	6,7
	373-387	IELEPPFGDSYIV	6,7
	377-391	PPFGDSYIVVGRG	6,7
	381-395	DSYIVVGRGEQQI	13,3
	385-399	VVGRGEQQINHHW	6,7
	389-403	GEQQINHHWHKSG	6,7
	393-407	INHHWHKSGSSIG	6,7
	413-427	RGAQRLAALGDTA	6,7
	417-431	RLAALGDTAWDFG	13,3
	425-439	AWDFGSVGGVFTS	6,7
	429-443	GSVGGVFTSVGKA	13,3
	433-447	GVFTSVGKAIHQV	13,3

[a]Amino acid position within the protein. [b]Percentage of responders recognizing a specific peptide.

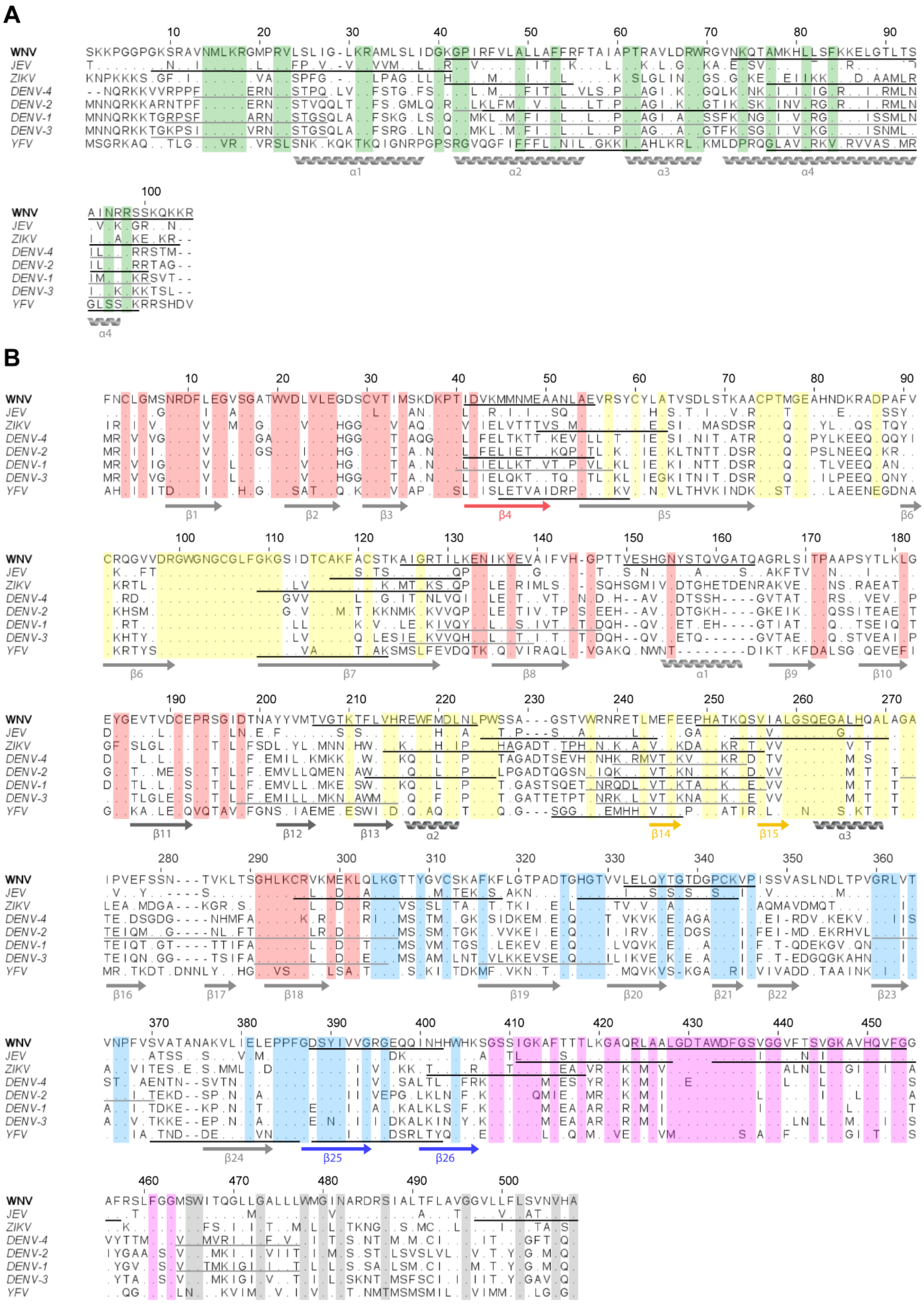

Figure S2. Multiple Sequence Alignment of C and E Proteins from Mosquito-borne Flaviviruses. Capsid **(A)** and envelope protein **(B)** sequences from WN (GenBank DQ211652), JE (GenBank D90194), Zika (GenBank KJ776791), DEN 1–4 (GenBank AF226687, M29095, DQ863638, GQ398256) and YF (GenBank CAA27332) viruses were aligned using CLUSTAL omega. Experimentally identified epitopes from WN virus and those from JE (46), Zika (23), DEN (39, 40), and

YF (22) are underlined in black. Additional epitopes from DEN (44, 45) are underlined in gray. Sequence elements with 90% amino acid identity across all flaviviruses analysed are highlighted (C: green; E: DI-red, DII-yellow, DIII-blue, stem-purple, transmembrane domain-gray). Positions of α-helices and ß-sheets as found in WN virus C (PDB 1SFK) (35) and E (PDB 2I69) (25) are shown below the alignment. The secondary structures of the common epitope regions (Figures 3 and 4) are highlighted (E: DI-red, DII-yellow, DIII-blue).

Table S2. HLA Class II in West Nile Virus-infected Individuals

	Haplotypes												
**Patient**	**DQA1**	**DQB1**	**DRB1**	**DQA1**	**DQB1**	**DRB1**	**DPA1**	**DPA1**	**DPB1**	**DPB1**	**DRB3**	**DRB4**	**DRB5**
501	03:03	03:01	04:07	01:02	06:02	15:01	01:03	01:03	04:01	02:01		01:03	01:01
502	03:01	03:02	04:04	05:05	03:01	11:04	01:03	01:03	04:02	02:01	02:02	01:03	
503	01:02	06:02	15:01	02:01	02:02	07:01	01:03	01:03	04:01	04:01		01:03	01:01
504	01:01	05:01	01:02	03:03	03:01	04:01	01:03	01:03	20:01	04:02		01:03	
505	01:05	05:01	10:01	05:05	03:01	11:01	01:03	02:01	10:01	04:01	02:09		
506	01:01	05:01	01:01	03:03	03:01	04:01	01:03	01:03	04:01	04:01		01:03	
507	02:01	02:02	07:01	05:05	03:01	11:04	01:03	02:01	09:01	04:01	02:02	01:03	
508	01:01	05:01	01:01	05:05	03:01	11:04	01:03	01:03	04:02	04:02	02:02		
509	01:01	05:01	01:01	05:05	03:01	11:04	01:03	01:03	02:01	02:01	02:02		
510	05:01	02:01	03:01	05:01	02:01	03:01	01:03	01:03	04^{new}	02^{new}	01:01, 02:02		
511	05:01	02:01	03:01	05:05	03:01	11:04	01:03	01:03	04:02	04:01	02:02		
512	01:02	06:03	13:01	01:03	06:04	13:02	01:03	01:03	04:02	02:01	02:02, 03:01		
513	01:02	06:02	15:01	04:01	04:02	08:04	01:03	01:03	06:01	04:01			01:01
514	01:01	05:01	01:01	02:01	02:02	07:01	01:03	02:02	05:01	04:02		01:01	
515	02:01	03:03	07:01	06:01	03:01	08:03	01:03	01:03	04:01	04:01		01:03N	
516	02:01	02:02	07:01	03:03	03:01	04:01	01:03	01:03	04:01	04:01		01:01	
517	01:02	06:02	15:01	05:05	03:01	11:04	01:03	02:01	17:01	03:01	02:02		01:01
518	03:01	03:02	04:01	05:05	03:01	11:03	01:03	01:03	04:02	04:01	02:02	01:03	
519	02:01	02:02	07:01	01:07Q	05:03	14:54	01:03	01:03	04:01	02:01	02:02	01:01	
520	01:02	06:02	15:01	05:01	02:01	03:01	01:03	01:03	04:02	04:01	02:02		01:01
521	01:01	05:01	01:02	01:04	05:03	14:54	01:03	02:01	14:01	04:01	02:02		
522	03:03	03:01	04:01	05:05	03:01	11:01	01:03	01:03	04:01	04:01	02:02	01:03	
523	01:02	05:02	16:01	01:03	06:03	13:01	01:03	01:03	04:01	04:01	02:02		02:02
524	01:02	05:02	16:01	05:05	03:01	11:04	01:03	01:03	04:02	02:01	02:02		02:02
525	03:01	03:02	04:01	05:05	03:01	11:03	01:03	02:06	05:01	04:01	02:02	01:03	
526	02:01	02:02	07:01	03:01	03:02	04:03	01:03	01:03	06:01	04:01		01:02, 01:03	
701	01:01	05:01	01:01	01:01	05:01	01:01	01:03	01:03	04:02	04:01	01:01, 02:02		
705	01:03	06:03	13:01	05:05	03:01	11:01	01:03	02:01	10:01	04:01			
712	01:02	05:02	16:01	05:05	03:01	11:04	01:03	01:03	06:01	04:01	02:02		02:02

Table S3. HLA Association of Dominant WN Virus Epitopes

Protein	WNV peptides		HLA II allele	Percentile rank	Frequency (%) of responders[b], (no. of alleles)[c]	Odds ratio (p-value)[d]
	aa[a] position	sequence				
C	41-55	KGPIRFVLALLAFFR	DRB4*01:03	0.57	27.3 (11)	15.24 (0.045)
	73-87	NKQTAMKHLLSFKKE	DRB5*01:01	0.28	40.0 (5)	35.0 (0.025)
	77-91	AMKHLLSFKKELGTL	DRB5*01:01	0.3	40.0 (5)	13.29 (0.042)
			DRB4*01:03	5.13		
	89-103	GTLTSAINRRSTKQK	DRB1*04:01	9.29	33.3 (6)	26.11 (0.037)
			DRB1*08:04	0.46		
	93-107	SAINRRSTKQKKRGG	DRB5*01:01	5.79	33.3 (6)	26.11 (0.037)
			DRB1*08:04	0.46		
E	41-55	IDVKMMNMEAANLAD	DRB1*01:02	0.18	28.6 (7)	26.11 (0.037)
			DRB1*04:01	0.57		
	209-213	KSFLVHREWFMDLNL	DQA1*01:02/ DQB1*05:02	0.57	30.0 (10)	22.8 (0.023)
			DRB1*07:01	9.04		
	245-259	PHATKQSVVALGSQE	DRB1*01:02	0.51	50.0 (4)	24.0 (0.042)
			DQA1*01:03/ DQB1*06:03	8.47		
	249-263	KQSVVALGSQEGALH	DRB1*11:04	6.37	30.0 (12)	18.53 (0.021)
			DRB1*01:02	1.1		
			DQA1*03:01/ DQB1*03:02	5.21		
	381-395	DSYIVVGRGEQQINH	DRB1*11:01	1.15	66.7 (3)	88.3 (0.007)
	429-443	GSVGGVFTSVGKAIH	DRB1*01:02	4.77	30.0 (6)	26.11 (0.037)
			DRB1*03:01	7.08		
	433-447	GVFTSVGKAIHQVFG	DRB1*07:01	14.66	28.5 (7)	20.45 (0.052)

[a]Amino acid position of peptides in C or E proteins. [b]Percent HLA II allele-positive donors who recognize certain peptides. [c]Number of HLA II allele-positive donors [d]The Fisher´s exact test was used to calculate the significance (p-value) of association.
